# Changed Hub and Corresponding Functional Connectivity of Subgenual Anterior Cingulate Cortex in Major Depressive Disorder

**DOI:** 10.3389/fnana.2016.00120

**Published:** 2016-12-16

**Authors:** Huawang Wu, Hui Sun, Jinping Xu, Yan Wu, Chao Wang, Jing Xiao, Shenglin She, Jianwei Huang, Wenjin Zou, Hongjun Peng, Xiaobing Lu, Guimao Huang, Tianzi Jiang, Yuping Ning, Jiaojian Wang

**Affiliations:** ^1^Key Laboratory for NeuroInformation of Ministry of Education, School of Life Science and Technology, University of Electronic Science and Technology of ChinaChengdu, China; ^2^Department of Radiology, The Affiliated Brain Hospital of Guangzhou Medical University (Guangzhou Huiai Hospital)Guangzhou, China; ^3^Beijing Key Laboratory of Learning and Cognition, School of Education, Capital Normal UniversityBeijing, China; ^4^Institute of Biomedical and Health Engineering, Shenzhen Institutes of Advanced Technology, Chinese Academy of SciencesShenzhen, China; ^5^Sichuan Applied Psychology Research Centre, Chengdu Medical CollegeChengdu, China; ^6^School of Psychology and Sociology, Shenzhen UniversityShenzhen, China; ^7^Department of Psychiatry, The Affiliated Brain Hospital of Guangzhou Medical University (Guangzhou Huiai Hospital)Guangzhou, China; ^8^Department of Clinical Psychology, The Affiliated Brain Hospital of Guangzhou Medical University (Guangzhou Huiai Hospital)Guangzhou, China; ^9^Department of Neurology, The Affiliated Brain Hospital of Guangzhou Medical University (Guangzhou Huiai Hospital)Guangzhou, China

**Keywords:** major depressive disorder, hub, subgenual anterior cingulate cortex, functional connectivity strength, resting-state

## Abstract

Major depressive disorder (MDD) is one of the most prevalent mental disorders. In the brain, the hubs of the brain network play a key role in integrating and transferring information between different functional modules. However, whether the changed pattern in functional network hubs contributes to the onset of MDD remains unclear. Using resting-state functional magnetic resonance imaging (rs-fMRI) and graph theory methods, we investigated whether alterations of hubs can be detected in MDD. First, we constructed the whole-brain voxel-wise functional networks and calculated a functional connectivity strength (FCS) map in each subject in 34 MDD patients and 34 gender-, age- and education level-matched healthy controls (HCs). Next, the two-sample *t*-test was applied to compare the FCS maps between HC and MDD patients and identified significant decrease of FCS in subgenual anterior cingulate cortex (sgACC) in MDD patients. Subsequent functional connectivity analyses of sgACC showed disruptions in functional connectivity with posterior insula, middle and inferior temporal gyrus, lingual gyrus and cerebellum in MDD patients. Furthermore, the changed FCS of sgACC and functional connections to sgACC were significantly correlated with the Hamilton Depression Rating Scale (HDRS) scores in MDD patients. The results of the present study revealed the abnormal hub of sgACC and its corresponding disrupted frontal-limbic-visual cognitive-cerebellum functional networks in MDD. These findings may provide a new insight for the diagnosis and treatment of MDD.

## Introduction

Major depressive disorder (MDD) is a psychiatric disorder characterized by affective, cognitive and vegetative symptoms. Given its high prevalence, MDD has become one of the worldwide leading causes of disability and societal and familial burdens (Mathers and Loncar, 2006). The primary features of MDD are persistent, pervasive feelings of sadness, guilt and worthlessness. Patients with MDD showed an enhanced attention to, and memory for, negative emotional stimuli in MDD (Wenzlaff et al., 1988; Mogg et al., 1995; Fales et al., 2008). As the disease progresses, severe patients even commit suicide. Therefore, the early diagnosis and prevention of MDD can provide an important strategy to alleviate the symptoms of MDD.

Resting-state functional MRI (rs-fMRI), which primarily reflects ongoing spontaneous fluctuations in the human brain, can non-invasively investigate the functional coupling between brain regions to detect intrinsic functional changes and abnormal functional modules (Fox et al., 2006; Buckner et al., 2009; Power et al., 2011; Yeo et al., 2011; Wang et al., 2012, 2015a,b; Cole et al., 2014; Wang J. et al., 2016; Mears and Pollard, 2016). In the brain, a few highly connected and central regions, the so-called hub nodes, play a key role in the global topology of the brain’s network (Hagmann et al., 2008; Bullmore and Sporns, 2009). Hubs also play central roles in integrating diverse informational sources and supporting fast communication with minimal energy cost (Bassett and Bullmore, 2006; van den Heuvel and Sporns, 2013). In MDD patients, many previous studies have revealed disrupted interactions between different brain networks (Sheline et al., 2010; Diener et al., 2012). Therefore, whether the changed hub and altered functional connectivities of hubs contribute to the pathophysiology of MDD remains unknown.

Thus, we hypothesized that MDD patients have disrupted functional network hubs, i.e., decreased functional connectivity strength (FCS) compared with normal healthy controls (HCs). Using the rs-fMRI data in a group of 34 MDD patients and 34 gender-, age- and education level-matched HCs, we examined the potentially abnormal connectivity patterns of hubs characterized with degree centrality using a graph theory approach. We first calculated a voxel-wise FCS map for each subject to identify the abnormal hubs in MDD (Cole et al., 2010; Wang et al., 2014). Subsequently, we mapped the resting-state functional connectivity of the identified hubs to further reveal its corresponding disrupted functional networks in MDD.

## Materials and Methods

### Subjects

Thirty-four MDD patients and 34 HCs were recruited at the Department of Psychiatry at the Affiliated Brain Hospital of Guangzhou Medical University, and all participants were right-handed individuals of Han Chinese ancestry ranging in age between 18 and 46 years. The diagnosis of MDD was made according to the Structured Clinical Interview of DSM-IV (SCID) criteria. All patients had a score of at least 20 on the 24-item Hamilton Depression Rating Scale (HDRS). Twenty-three MDD patients were medication-free, defined as not taking antidepressants in the current episode, and the other 11 patients were administered a single dose of antidepressants. All HCs were screened using the SCID Non-Patient Edition to confirm the lifetime absence of Axis I illness and selected control subjects had no known history of psychiatric illness in any two lines of first to third degree biological relatives. All MDD patients and HCs were without a lifetime history of seizures, head trauma, serious medical or surgical illness, substance abuse or dependance, or contraindications for MRI. Potential participants were excluded if signs of gross abnormalities were detected on cerebral examination. Written informed consent was obtained from all subjects in accordance with the Declaration of Helsinki, and approval was obtained through the ethics committees of the Affiliated Brain Hospital of Guangzhou Medical University. The demographic and psychological characteristics of the samples are summarized in Table 1.

**Table 1:** **Demographics and clinical characteristics of the subjects used in the present study**.

Subjects	MDD patients	Healthy controls	*P* value
Number of subjects	34	34	
Age (mean ± SD)	29.88 ± 7.070	29.71 ± 7.086	0.918
Gender (male: female)	14:20	15:19	0.806
Years of education	13.20 ± 3.540	14.18 ± 2.167	0.171
(mean ± SD)
HDRS scores (mean ± SD)	32.91 ± 7.025
Age of onset (years)	25.44 ± 7.386
Duration of illness (months)	53.41 ± 61.185
Episodes (*n*, patients)
First	21
Recurrence	13
Family history of MDD	11
(*n*, patients)
Medication (*n*, patients)
Medication-free	23
On-medication	11
SSRI antidepressant	8
SNRI antidepressant	1
NaSSA antidepressant	1
Melatonergic antidepressant	1

*Note: Pearson’s chi-squared test was used for gender comparison. Two-sample *t*-tests were used for age, education comparisons. HDRS, Hamilton Depression Rating Scale scores. SSRI, selective serotonin reuptake inhibitor; SNRI, serotonin norepinephrine reuptake inhibitor; NaSSA, noradrenergic and specific serotonergic antidepressant*.

### fMRI Data Acquisition

MRI data was acquired on a 3.0 Tesla MR imaging system (Achieva X-series, Philips Medical Systems, Best, Netherlands) with an eight-channel SENSE head coil, in the Department of Radiology, the Affiliated Brain Hospital of Guangzhou Medical University, Guangzhou, China. Tight but comfortable foam padding was used to reduce head motion, and earplugs were used to muffle scanner noise. The participants were instructed to rest with their eyes closed during scanning. No participant reported falling asleep during the scan when routinely asked immediately after scanning. rs-fMRI images were acquired using a gradient-echo echo-planar imaging (GRE-EPI) sequence sensitive to blood oxygenation level-dependent (BOLD) contrast. The main parameters include repetition time (TR) = 2000 ms, echo time (TE) = 30 ms, flip angle (FA) = 90°, matrix = 64 × 64, field of view (FOV) = 220 mm × 220 mm, slice thickness = 4 mm with inter-slice gap = 0.6 mm, 33 interleaved axial slices and 240 time points.

### fMRI Data Preprocessing

The rs-fMRI data was preprocessed using SPM8 software with DPARSF version 2.3^1^. The first 10 volumes were discarded to facilitate magnetization equilibrium. After slice timing, the corrected time series was realigned to the first volume for head motion correction. The data was discarded if the head-movement exceeded 1.5 mm of translation or 1.5° of rotation in any direction. The fMRI images were normalized to the EPI template in MNI space and resampled to a 3 × 3 × 3 mm^3^ voxel size. Next, the functional images were detrended and six motion parameters, white matter and cerebrospinal fluid signals were regressed out. Because the whole-brain signal regression will exaggerate anti-correlation, the global signal was not regressed to ensure that the obtained results were reliable. Subsequently, the functional images were filtered with a temporal band-path of 0.01–0.1 Hz. Finally, considering a recent study showing that motion influenced measures of functional connectivity (Power et al., 2012), the time course for each run was “scrubbed” by eliminating the bad images, captured before two time points and after one time points, which exceeded the pre-set criteria (frame displacement: FD, FD < 0.5) for excessive motion. The FD values for all subjects were below 0.3; thus, no frame was deleted. For the following functional connectivity analyses, the fMRI data was smoothed using a Gaussian kernel of 6 mm full-width at half maximum (FWHM).

### Whole Brain Voxel-Wise Functional Connectivity Strength Calculation

For each subject, the FCS value was calculated for each voxel. The functional connectivity calculation was constrained within a binary gray matter mask created by thresholding the gray matter probability template with 0.2 (Wang et al., 2014). First, the time series of the seed voxel and all other voxels of the resting brain were extracted, and Pearson’s correlation coefficients between the time series of the seed voxel and that of all other voxels were calculated. Next, a threshold of 0.2 was set to remove weak connections that may arise from signal noise based on a previous study (Wang et al., 2014). Next, the FCS was calculated by averaging the correlation coefficients higher than this threshold over the whole brain. The entire process was repeated for all other voxels, and a FCS map for each subject was obtained. Subsequently, the FCS map was converted to *z* scores. Such a FCS metric is referred to as the “degree centrality” of weighted networks in terms of graph theory (Buckner et al., 2009; Zuo et al., 2012). The FCS map was subsequently spatially smoothed with a 6 mm FWHM Gaussian kernel and subjected to statistical analysis.

### Statistical Analyses

First, one-tailed one-sample *t*-tests were used to identify the brain hub regions in MDD and HC. The significance was determined using the false discovery rate (FDR) correction method with *p* < 0.05. A mask was created by combining the one-sample *t*-test results in both MDD and HC groups and used for the subsequent between group statistical analyses.

Next, a two-tailed two-sample *t*-test (gender, age and education as covariates) was performed to compare the FCS maps between the MDD and HC groups to reveal the disrupted brain hub regions. The significance was determined using FDR correction method with *p* < 0.05. To exclude the drug effect, we re-analyzed the medication-free subjects’ data using the same procedures.

### Functional Connectivity Analyses

To identify the changed functional connectivity with the identified hub which has significant differences in degree centrality between MDD and HC, the whole brain functional connectivity analysis of the hub regions was performed. We first extracted the mean time series of the identified hub region. Then, the strength of the functional connectivity was measured through Pearson’s correlations between the averaged time series of the hub region and voxels in the rest of the brain. Subsequently, the Fisher’s *z* transformation was applied to normalize the original correlation maps, and a two-sample *t*-test (gender, age and education as covariates) was performed to determine areas with significantly different functional connectivity to the hub regions between MDD and the HCs. Because the FDR method is too strict for whole brain functional connectivity analyses, the significance in the present study was determined using the Gaussian random field (GRF) correction *p* < 0.05 (*z* > 2.3). To exclude the drug effect, we re-analyzed the medication-free subjects’ data using the same procedures.

### Correlation Analyses

To determine the relationship between FCS, resting-state functional connection and HDRS scores, correlation analyses were performed between the mean FCS in changed hub region, the mean functional connections of the functionally altered brain areas to subgenual anterior cingulate cortex (sgACC) and the HDRS. The significance was set at *p* < 0.05.

## Results

### Demographics and Clinical Characteristics

The demographics and clinical characteristics of the subjects used in current study are presented in Table 1. No significant differences in gender (*p* = 0.806), age (*p* = 0.918) and education level (*p* = 0.171) were observed between MDD and HC groups.

### Hubs in MDD and HC

**Figure 1:**
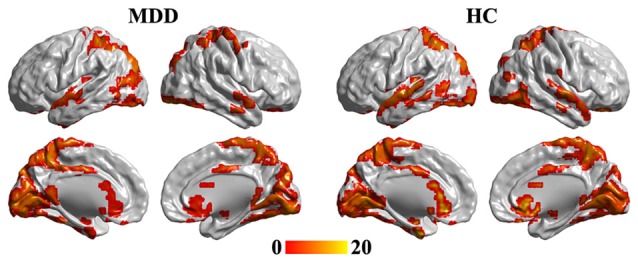
**One-tailed one-sample *t*-tests were used to identify the brain hub regions in MDD and HC.** The significance was determined using false discovery rate (FDR) correction method with *p* < 0.05. The left panel is the hubs in MDD patients, and the right panel is the hubs in HC group. Color bar is the *t* value of one-sample *t*-test analysis. MDD, major depressive disorder; HC, healthy control.

One-sample *t*-tests were used to identify the hubs in MDD and HC groups. The hub brain areas were primarily detected in the superior temporal gyrus, occipital gyrus, fusiform gyrus, intraparietal sulcus, superior parietal cortex, precuneus, middle cingulate cortex, caudate, and sgACC and cerebellum (Figure 1).

### Changed Hubs in MDD

**Figure 2:**
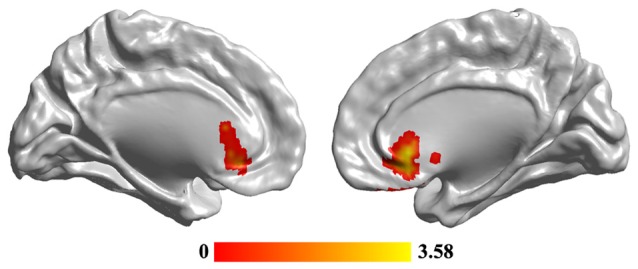
**The reduced functional connectivity strength (FCS) of subgenual anterior cingulate cortex (sgACC) in MDD patients.** Two-sample *t*-test was used to compare the FCS maps between HCs and MDD patients. The significance was determined using FDR correction method with *p* < 0.05. The hub analysis identified significantly decreased FCS in sgACC in MDD patients.

Statistical analyses identified significant differences in FCS maps between MDD and the HC, and a significant decrease of FCS in MDD was observed in the sgACC (peak MNI coordinate: [6, 27, −3], 246 voxels; Figure 2). In addition, we re-analyzed the medication-free subjects’ data and obtained the similar results (Figure S1 in Supplementary Material).

### Disrupted Functional Networks to sgACC

**Figure 3:**
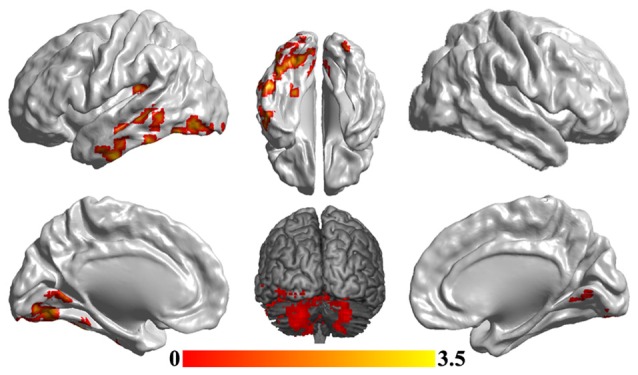
**Disrupted functional networks to sgACC in MDD patients.** Two-sample *t*-tests were used to identify the significant differences in functional connectivity between MDD and HC groups. The significance was determined using Gaussian random field (GRF) correction *p* < 0.05 (*z* > 2.3). The functional connectivity analyses identified disrupted functional connections of posterior insula, middle and inferior temporal gyrus, lingual gyrus and cerebellum to sgACC.

To reveal the disrupted functional networks to the identified hub region, whole brain functional connectivity analyses for sgACC were performed, identifying decreased connections to posterior insula, middle and inferior temporal gyrus, lingual gyrus and cerebellum in MDD compared with HCs (Figure 3 and Table 2). In addition, we re-analyzed the medication-free subjects’ fMRI data and obtained the similar differences in functional connectivity patterns (Figure S1 in Supplementary Material).

**Table 2:** **Regions showing differences in functional connectivity between major depressive depression (MDD) patients and healthy controls (HCs)**.

Comparison	Regions	L/R	Peak MNI coordinate (*x y z*)	Maximal *t*-value	Cluster size (voxels)
HC > MDD	Cerebellar Tonsil	L	−15 −42 −48	3.3318	89
	Cerebelum_Crus2	L	−27 −87 −45	3.3173	202
	Cerebelum_Crus1_2	R	30 −90 −33	3.1536	68
	Cerebelum_8	R	6 −63 −36	2.8497	61
	Vermis_4_5	R	3 −66 −3	3.5774	101
	Middle temporal gyrus	L	−57 −42 −6	3.3042	67
	Posterior insula	L	−36 −36 6	3.1527	57

*Note: L, left; R, right*.

### Correlation Analyses

**Figure 4:**
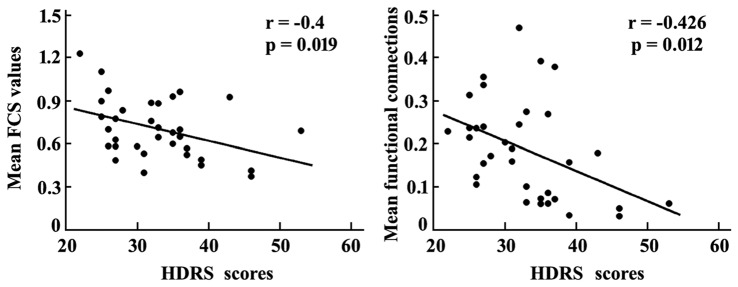
**Correlation analyses between the mean FCS in sgACC, mean resting-state functional connections of the brain areas with altered functional connectivities with sgACC and the HDRS scores were performed to determine the relationship between the neural indices and behavior in MDD patients.** The significant was set at *p* < 0.05. HDRS, Hamilton Depression Rating Scale.

Correlation analyses revealed that the mean FCS value in the sgACC was significantly correlated with the HDRS scores in MDD patients (*r* = −0.4, *p* = 0.019). In addition, a significant association between the mean resting-state functional connections of the brain regions with changed functional networks to sgACC and HDRS scores in MDD patients was also identified (*r* = −0.426, *p* = 0.012; Figure 4).

## Discussion

In the present study, we investigated the changed hub of brain and corresponding functional alterations between MDD and HC participants. The hub analysis revealed decreased FCS in subgenual ACC (sgACC) in MDD. Subsequent functional connectivity analyses of sgACC revealed decreased functional connectivity with limbic, visual cognitive networks and the cerebellum.

The ACC plays an important role in emotion processing (Vogt, 2005; Etkin et al., 2011). The dorsal ACC plays a key role in emotion monitoring (Beck, 2008; Behrens et al., 2008; Pizzagalli et al., 2009), whereas the ventral ACC (particularly the sgACC) is primarily involved in emotion experience (Drevets et al., 2008; Groenewold et al., 2013). Structural and functional abnormalities in sgACC have been widely reported in MDD using neuroimaging methods. Structural magnetic resonance imaging (sMRI)-based studies have revealed that the gray matter volume of sgACC was reduced in MDD patients (Drevets et al., 1997, 1998; Botteron et al., 2002; Coryell and Young, 2005). In addition, resting-state overactivity in the sgACC was also observed in MDD patients compared with the HCs, indicating that the MDD patients showed greater activation (less deactivation; Drevets et al., 1992; Drevets, 2001; Fales et al., 2008). Recent studies using deep brain stimulation (DBS) on sgACC also revealed that stimulation of sgACC can effectively reverse symptoms in MDD (Mayberg et al., 2005). Furthermore, the anatomical connectivity analyses of patients with DBS treatment also suggested that the sgACC plays a more important role in antidepressant effect (Johansen-Berg et al., 2008). These findings suggested that sgACC acts as a hub for a MDD-related neural circuit. In the present study, using hub analyses method, we directly demonstrated that the abnormality of sgACC is the core of MDD-related brain networks. Correlation analyses further demonstrated that the mean FCS values in MDD were correlated with the symptoms of depression, suggesting that the changes in FCS in sgACC can predict the symptoms of depression and may serve as a biomarker for the diagnosis and treatment of depression. The findings for MDD were supported by a previous resting-state study, which also identified the changed hub of sgACC (Wang et al., 2014). In the present study, we identified the abnormal hub area of sgACC, and the other brain regions, such as the cerebellum, insula and lingual gyrus, only showed functional connectivity abnormalities to sgACC, suggesting that the abnormalities of other brain regions may result from sgACC dysfunction. These findings further supported the view that sgACC is the core of the changed brain network in MDD.

The disrupted functional connections between the sgACC and posterior insula, lingual gyrus and inferior temporal gyrus were revealed in the present study. The insula is an important limbic structure that participates in many cognitive functions, such as language, attention, emotion and interoception (Kelly et al., 2012). The posterior insula, which primarily connects to primary and secondary somatomotor cortices, is a critical site for interoception (Craig, 2002; Deen et al., 2011). A breakdown in this circuitry could potentially account for the somatic complaints in MDD (Cullen et al., 2009). The lingual gyrus and inferior temporal gyrus, which also showed decreased functional connectivity to sgACC in MDD, are parts of the visual recognition circuit. The visual recognition circuit primarily participates in object identification (Fujita, 2002) and affect identification (Ishai, 2008; Collins and Olson, 2014). The disrupted connectivity between visual cognitive circuit and sgACC suggested that the damaged modulation of sgACC on visual cognitive circuit in MDD might result in negative bias in affect identification.

In addition to somatosensory and visual cognitive circuits, we also identified decreased functional connections with cerebellum and middle temporal gyrus, which are two nodes of the default mode network (DMN; Raichle et al., 2001; Greicius et al., 2003; Wang C. et al., 2016). The cerebellum was initially proposed as the sole contributor to the planning and execution of movement, but an increasing number of studies have revealed that the cerebellum also participates in many other cognitive functions, such as language, attention and emotion processing (Strick et al., 2009; Leiner, 2010; Schmahmann, 2010; Buckner, 2013). Although previous studies have reported the functional abnormality of the cerebellum at resting-state in MDD, the role of the cerebellum in MDD remains unclear (Dichter et al., 2015). Previous studies have revealed that the overactivity of DMN in MDD patients suggests that the cerebellum and middle temporal gyrus contribute to behavioral disturbances in MDD, associated with rumination occurrence (Sheline et al., 2010; Nejad et al., 2013). The correlation analyses performed in the present study revealed a significant correlation between HDRS scores and functional connectivity between sgACC and visual recognition and cerebellum circuits. These findings indicated that the dysregulation of sgACC in the visual cognition network, cerebellum and middle temporal gyrus was closely associated with symptoms of MDD.

There are some limitations in the present study. First, in the present study, only 34 MDD patients were used to investigate changes of the functional hubs. Thus, these findings should be further validated in a larger sample. Second, some MDD patients were on medication or showed recurrence episodes, and whether drugs or recurrence affected these findings should also be examined in first episode and drug naive MDD patients. Third, the duration of disease greatly varied in MDD patients, which may also affect the results.

## Conclusion

In conclusion, the present study assessed the abnormality of hubs and the disrupted resting-state functional connectivity in MDD. The decreased cortical hub of sgACC was identified in MDD. Whole brain functional connectivity analyses revealed decreased functional connections in posterior insula, lingual gyrus, middle and inferior temporal gyrus and cerebellum to sgACC in MDD. These findings further supported the core role of sgACC in MDD, and the dysregulation of sgACC in other brain regions may contribute to the onset of MDD. Thus, these findings may facilitate the future diagnosis and DBS-related therapy for MDD.

## Author Contributions

JW, TJ and YN designed and supervised the study; HW, JH and WZ collected the data; and HW, JW, HS, JX, YW and JX analyzed the data. HW, HS, CW and JW drafted the manuscript. All authors discussed the results and commented on the manuscript.

## Conflict of Interest Statement

The authors declare that the research was conducted in the absence of any commercial or financial relationships that could be construed as a potential conflict of interest.

## Footnotes

^1^www.restfmri.net/forum/DPARSF
