# Supplementary material for: Changed Hub and Corresponding Functional Connectivity of Subgenual Anterior Cingulate Cortex in Major Depressive Disorder
Source: Front Neuroanat. 2016 Dec 16;10:120. doi: 10.3389/fnana.2016.00120 (PMC5159433; doi:10.3389/fnana.2016.00120)

Figure S1. We re-analyzed the functional connectivity strength (FCS) and corresponding functional connectivity patterns in 23 medication-free patients to determine whether our findings from the 34 major depressive disorder (MDD) patients are drug effect. The results obtained from 23 medication-free MDD patients are similar to the findings from all the 34 MDD patients suggesting our findings are not the drug effect.

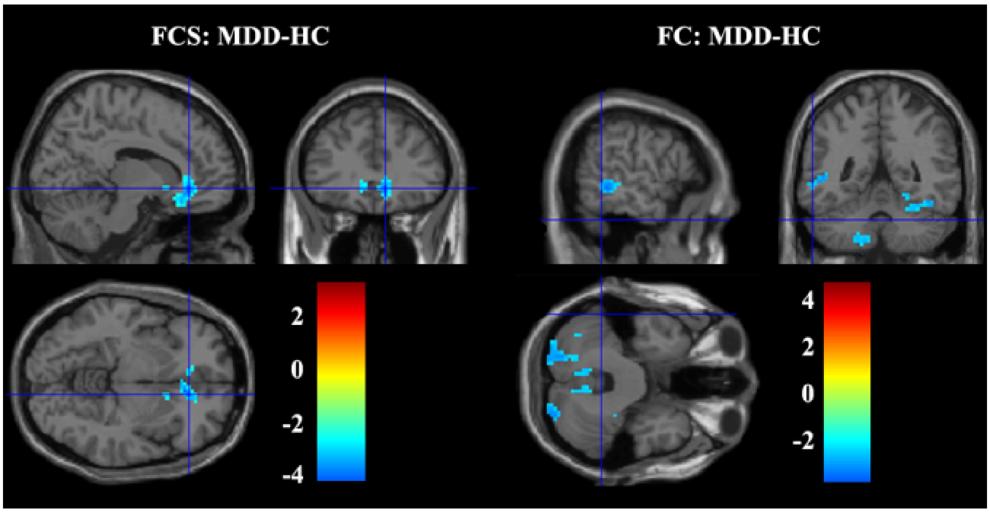

Supplement: Supplementary file 1 [file Presentation_1.pdf]
